# Detrimental role of prolonged sleep deprivation on adult neurogenesis

**DOI:** 10.3389/fncel.2015.00140

**Published:** 2015-04-14

**Authors:** Carina Fernandes, Nuno Barbosa F. Rocha, Susana Rocha, Andrea Herrera-Solís, José Salas-Pacheco, Fabio García-García, Eric Murillo-Rodríguez, Ti-Fei Yuan, Sergio Machado, Oscar Arias-Carrión

**Affiliations:** ^1^Faculty of Medicine, University of PortoPorto, Portugal; ^2^Laboratory of Neuropsychophysiology, Faculty of Psychology and Education Sciences, University of PortoPorto, Portugal; ^3^School of Health Technologies, Polytechnic Institute of PortoPorto, Portugal; ^4^School of Accounting and Administration of Porto, Polytechnic Institute of PortoPorto, Portugal; ^5^Unidad de Trastornos del Movimiento y Sueño, Hospital General Dr. Manuel Gea González/Instituto de Fisiología Celular, Universidad Nacional Autónoma de MéxicoMexico City, Mexico; ^6^Instituto de Investigación Científica, Universidad Juárez del Estado de DurangoDurango, Mexico; ^7^Departamento de Biomedicina, Instituto de Ciencias de la Salud, Universidad VeracruzanaXalapa, Mexico; ^8^División Ciencias de la Salud, Laboratorio de Neurociencias Moleculares e Integrativas, Escuela de Medicina, Universidad Anáhuac MayabMérida, México; ^9^School of Psychology, Nanjing Normal UniversityNanjing, China; ^10^Panic and Respiration, Institute of Psychiatry of Federal University of Rio de JaneiroRio de Janeiro, Brazil; ^11^Physical Activity Neuroscience, Physical Activity Sciences Postgraduate Program, Salgado de Oliveira UniversityNiterói, Brazil

**Keywords:** sleep, adult neurogenesis, hypnotic drugs, antidepressants, circadian rhythms, hippocampus

## Abstract

Adult mammalian brains continuously generate new neurons, a phenomenon called adult neurogenesis. Both environmental stimuli and endogenous factors are important regulators of adult neurogenesis. Sleep has an important role in normal brain physiology and its disturbance causes very stressful conditions, which disrupt normal brain physiology. Recently, an influence of sleep in adult neurogenesis has been established, mainly based on sleep deprivation studies. This review provides an overview on how rhythms and sleep cycles regulate hippocampal and subventricular zone neurogenesis, discussing some potential underlying mechanisms. In addition, our review highlights some interacting points between sleep and adult neurogenesis in brain function, such as learning, memory, and mood states, and provides some insights on the effects of antidepressants and hypnotic drugs on adult neurogenesis.

## Introduction

Thousands of new neurons are daily added to the adult brain of different species, including humans (Yuan et al., 2014), and this constant lifelong generation implies significant structural changes (Eriksson et al., 1998; Curtis et al., 2007). Neurogenesis is involved in numerous brain processes but its association with sleep deprivation was only recently addressed. Previous research has focused on how generation and development of new neurons are affected by sleep loss. Short periods of sleep deprivation do not significantly alter the basal rate of cell proliferation, whereas long periods result in a decrease of cell proliferation and survival in the hippocampus (Mirescu et al., 2006). In this review, we will discuss the possible effects of sleep deprivation on the natural course of neurogenesis, and address the potential connections between sleep and neurogenesis that may influence other cognitive and neuropsychobiological functions, such as learning, memory, and mood disorders.

## Adult Neurogenesis

Neurogenesis includes the generation, proliferation, fate specification and integration of new functional neurons in the existing neural circuits from undifferentiated progenitor cells. Traditionally, it was believed that neurogenesis mainly occurred during the embryonic stages of the central nervous system (CNS), ending permanently at puberty (Ming and Song, 2005; Meerlo et al., 2009).

New findings, based on techniques such as [^3^H]-thymidine autoradiography (Sidman et al., 1959) and 5-bromo-2^′^-deoxyuridine (BrdU; Nowakowski et al., 1989), which mark cells in S phase of mitosis, electronic microscopy (Kaplan, 1977, 1981, 1984, 1985) and combining retroviral-based lineage tracing with electrophysiological methods (Sanes et al., 1986; Price et al., 1987) revealed the continuous adult neurogenesis and synaptic integration. Newborn neurons were found in certain CNS regions of birds (Goldman and Nottebohm, 1983), rats (van Praag et al., 1999), monkeys (Kornack and Rakic, 1999), and humans (Eriksson et al., 1998; Curtis et al., 2007), throughout life.

Neurogenesis occurs in specific areas of the CNS, namely in the subventricular zone (SVZ), lining the wall of the lateral ventricles, and in the subgranular zone (SGZ) of the hippocampal dentate gyrus (Alvarez-Buylla and Lim, 2004). The neurogenic behavior of these areas appears to be determined by signals of endothelial cells and astrocytes (Alvarez-Buylla and Lim, 2004).

Similarly, a cohort of glucogenic and neurogenic signals (Lim et al., 2000) regulates the underlying molecular mechanisms of neuronal differentiation, fate specification (Ming and Song, 2005; Arias-Carrión et al., 2007; Yuan and Arias-Carrion, 2011; Höglinger et al., 2014) and migration of new generated cells (Hu et al., 1996; Conover et al., 2000; Murase and Horwitz, 2002; Bolteus and Bordey, 2004). Newly formed neurons in the SVZ reach the olfactory bulb, by the rostral migratory stream (Saghatelyan et al., 2004), forming granule and periglomerular neurons. These neurons establish dendro-dendritic synapses with tufted cells (Abrous et al., 2005), beginning a maturation process by receiving GABAergic and glutamatergic synaptic inputs (Belluzzi et al., 2003). When maturate, the granule neurons secrete GABA, while periglomerular neurons secrete GABA and dopamine (Wang et al., 2000; Arias-Carrión et al., 2007). In the dentate gyrus, newborn neurons reach the anterior layer of the granule cells (Hastings and Gould, 1999), maintaining their neuronal maturation and synaptogenesis over several months (van Praag et al., 2002). It is believed that these neurons receive initially GABAergic and later glutamatergic inputs (Ming and Song, 2005). Once mature, most of these neurons secrete glutamate, while a small population releases GABA (Wang et al., 2000).

Adult neurogenesis is modulated by intrinsic and extrinsic factors (**Table 1**), being possible that several modulating factors remain unknown (Kempermann, 2002).

**Table 1:** Regulatory factors of adult neurogenesis.

Regulatory factors	Implications on adult neurogenesis	Potential mechanisms	Reference
**Genetic Background**	Influence neurogenesis in the SGZ		Kempermann and Gage (2002)
**Gender**	Cell proliferation in the SGZ is higher in females	Ovarian hormone levels (estrogen)	Tanapat et al. (1999)
**Aging**	Decrease cell proliferation in the SVZ and SGZ	Increased levels of corticosteroids	Kuhn et al. (1996), Cameron and McKay (2001), Jin et al. (2003), Enwere et al. (2004)
**Hormones**
Estrogen	Stimulate neurogenesis in the SGZ		Tanapat et al. (1999)
Corticosterone	Decrease neurogenesis in the SGZ	Activation of the hypothalamic-pituitary-adrenal axis (HPA)	Tanapat et al. (1999)
Prolactin	Stimulate neurogenesis in the SGZ and SVZ	Activation of the extracellular signal-regulated kinase 5	Tanapat et al. (1999), Wang et al. (2013)
**Neurotransmitters**
Dopamine	Decrease neurogenesis	Dopamine D2L receptors	Kempermann (2002), Radley and Jacobs (2002), Banasr et al. (2004)
Serotonin	Increase neurogenesis	Serotonin-1A receptors	
Acetylcholine	Decrease neurogenesis		
Glutamate	Decrease neurogenesis	Metabotropic glutamate receptors, NMDA receptors	
Nitric oxide	Decrease neurogenesis		
**Enriched Environments**	Increase the survival of newborn neurons in the SGZ	Peripheral vascular endothelial growth factor	Barnea and Nottebohm (1994), Kempermann et al. (1997), Nilsson et al. (1999), Brown et al. (2003)
**Physical exercise**	Promote cell proliferation and survival in the SGZ	Peripheral vascular endothelial growth factor	van Praag et al. (1999), Cao et al. (2004)
**Physical and psychosocial stress**	Decrease cell proliferation and amount of new neurons in the SGZ	Activation of the HPA	Gould and Tanapat (1999), Duman et al. (2001)
**Antidepressants**	Increased neurogenesis in the SGZ	Brain-derived neurotrophic factor	Eisch et al. (2000)
**Drugs of abuse**	Decrease cell proliferation and survival in the SGZ		Eisch et al. (2000), Crews et al. (2003)

## Sleep-Wake Cycle Regulation

Sleep and wakefulness are rhythmic behaviors regulated by circadian rhythms (Murillo-Rodríguez et al., 2009; Arias-Carrión et al., 2011). Wakefulness is a state of full manifestation of perceptual-sensory and voluntary motor activity (Siegel, 2001; Murillo-Rodríguez et al., 2009). Sleep is characterized by a rapid reversibility, reduced motor activity, responsiveness and metabolism (Siegel, 2009) and can be divided into distinct stages, cyclically repeated: the rapid eye movement sleep (REMS) and non-REM sleep (NREMS; Murillo-Rodríguez et al., 2009; Arias-Carrión et al., 2011; Ruehland et al., 2011). There are evidences that sleep plays an essential role in learning and memory (Keisler and Willingham, 2007; Song et al., 2007; Rasch et al., 2008; Rickard et al., 2008; Sheth et al., 2009), regulation of the immune system (Imeri and Opp, 2009; Opp, 2009), reversal of oxidative stress (Eiland et al., 2002) and neurogenesis (Guzman-Marin et al., 2005).

The regulation of sleep–wake cycles can be described by the two-process model, in which one process reflects homeostatic sleep drive, whereas the other is associated to the output of a circadian pacemaker. Combined, the two processes determine the beginning and end of sleep phase: when homeostasis increases above a certain threshold, sleep is triggered; when it drops below a certain threshold, wakefulness occurs. The circadian rhythm is thought to be a daily oscillatory modulator of the two thresholds (Daan et al., 1984).

Internally, the sleep–wake rhythm is centrally coordinated by an endogenous circadian clock, placed in the suprachiasmatic nucleus (SCN) of the anterior hypothalamus (Maywood et al., 2006). The neurons of the SCN are circadian oscillators that form functional pacemakers (Saper et al., 2001; Cheng et al., 2002). The timing of their oscillations is determined by an intrinsic cellular rhythmicity, which lasts 24 h, even in the absence of external inputs (Moore et al., 2002) such as light, feeding patterns, and social environment (Mueller et al., 2013).

## Sleep and Neuronal Assemblies

Despite the existence of a SCN biological clock (Maywood et al., 2006), Krueger et al. (2008) suggest that the global coordination of the NREMS results from an emergent property of local coupled processes in neural networks.

Cortical columns, also called neuronal assemblies, are examples of anatomically well-defined neural networks. It is believed that neuronal assemblies are the basic units of the brain processing during wakefulness, oscillating between functional states such as wake-like and sleep-like states (Rector et al., 2005). During sleep, most neuronal assemblies are in a sleep-like state and, during wakefulness, most of them are in a wake-like state. However, neuronal assemblies in sleep-like state can occur throughout all wakefulness phase and, oppositely, neuronal assemblies in wake-like state may occur during the whole-sleep phase (Krueger et al., 2008). The model of neuronal assemblies suggests that the synchrony between assemblies is a consequence of electrical and hormonal interactions that arise between them. Therefore, networks, that naturally occur with a weak interaction between their components, appear to exhibit emergent properties (collective behaviors that are not easily visible from the individual features of each assembly) that may achieve the synchronization and the strength required to change states (Roy et al., 2008).

This model postulates that states vary throughout time due to the individual behavior of each network, the interaction with other assemblies and threshold-based transitions (Krueger et al., 2008). Neuronal assemblies are quickly driven to sleep when individual neural networks enter in the sleep-like state. The strength of this response is proportional to the strength of the connections between individual neuronal assemblies. At the same time, if the circadian clock indicates that the body should be sleeping, the response of neuronal assemblies increases faster, triggering sleep. Experiments *in silico* suggest that neuronal over-stimulated assemblies will quickly enter in the sleep-like state, inducing surrounding neuronal assemblies to enter in the same state and leading to the whole-animal sleep. Thus, this model considers both the evolution of a global state of sleep and the emerging features of individual networks.

Sleep is currently seen as being imposed by the brain and regulated by an endogenous biological clock. However, this paradigm does not address many well-known phenomena of sleep, such as sleep inertia, restoring peak performance during sleep, homeostatic mechanisms of sleep, insomnia, somnolence or fatigue. The model of neuronal assemblies is more flexible, being easier to propose explanations for some sleep phenomena. For instance, sleep inertia may be a manifestation of some neuronal assemblies that remain in sleep-like state, after a sufficient number of neuronal assemblies are in wake-like state. In insomnia, some neuronal assemblies can be asleep, while others remain awake. The degree of sleepiness or the speed and accuracy of performance may be dependent on the fraction of neuronal assemblies that remain in wake-like state or sleep-like state. Brain imaging techniques evidenced that patients with insomnia display specific activation of wakefulness in some brain areas, while other areas have characteristics of sleep activity (Nofzinger et al., 2006).

To date, the model of neuronal assemblies does not provide answers on how many assemblies are needed to enter in a sleep-like state. However, similar limitations exist in the current paradigm of sleep regulation, which proposes a top–down imposition of sleep on the brain by regulatory circuits, not specifying which and how many areas need to be activated to induce sleep. Despite the insufficient understanding of the mechanisms of sleep, this model provides an evolutionary conceptual framework for further researches (Krueger et al., 2008).

## Sleep Effects on Adult Neurogenesis

### Seasonal Changes of Neurogenesis

There is a correlation between sleep and neurogenesis across lifespan, since cell proliferation is maximal during early development stages, when daily amounts of sleep are higher. In addition, seasonal variability in neurogenesis and in sleep expression are associated in some species that migrate or hibernate (Mueller et al., 2013). In adult birds, for instance, neurogenesis and sleep patterns are noteworthy due to their marked variations in annual rates.

Tramontin and Brenowitz (2000) have shown that, in songbirds, the breeding season is anticipated by an increase in neuronal number, size and spacing in brain regions responsible for controlling song. According to the authors, this increase is related to seasonal changes in song production and learning and is induced by a vernal enhance in circulating sex steroids.

Claytona et al. (1997) analyzed the seasonal differences in hippocampal volume of two parasitic species of cowbirds (*M. bonariensis* and* M. rufoaxillaris*), reporting a decrease in the hippocampal volume during the non-breeding season in both species. They justified this decrease with seasonal changes in spatial memory demands. During the breeding season, an enlarged hippocampus is important for hosts’ nests location. In contrast, during the non-breeding season, the lack of this demand is associated with a decrease in the relative hippocampal volume.

Barnea and Nottebohm (1994) injected adult black-capped chickadees with [H^3^]-thymidine and released and recaptured birds after 6 or more weeks. Newly formed neurons appeared in the hippocampus throughout the year, however, with a marked peak during the fall (October). This peak was halved in captive chickadees. Despite the addition of new neurons, they found no differences in the number of neurons along seasons. This evidence suggests that new neurons were born in different periods of the year, lived for few months, occurring, subsequently, neuronal loss. The authors hypothesized that new neurons added during the fall are part of an important process of new spatial memories acquisition, which is particularly important at this time of the year due to the food hoarding. They proposed that storing memories turned these neurons unviable for future processing, requiring them to be replaced.

Smulders et al. (2000) found similar results. They found a seasonal variation in hippocampal volume of black-capped chickadees in the fall, mainly associated to an increased number of neuronal and glial cells. Later, the number of neurons and glial cells decreased in parallel with the reduction of hoarding behavior. The authors suggested that this increase in the number of neurons and glial cells may provide a larger neural network to process information regarding more locations, a necessary ability to efficiently distribute food during the fall. They further suggested that this increase may be produced by seasonal signals inherent to the fall approximation, while its maintenance during hoarding season could be dependent of experience.

Studies with different species of birds found similar results: an increase in hippocampal volume during the fall, correlated with an increase of cell proliferation. This structural change appears to be associated with seasonal demands of spatial memory (Barnea and Nottebohm, 1994; Claytona et al., 1997; Smulders et al., 2000) and song production and learning (Tramontin and Brenowitz, 2000), in response to the breeding and hoarding seasons. Previous studies suggested that sleep plays, in birds, a crucial role on spatial memory and song learning processes (Gobes et al., 2010). Controversially, sleep is markedly reduced in the migratory season (spring and fall), when spatial memory and song learning processes are more acute. This reduction in amount of sleep during the migratory season does not affect memory and learning of birds. However, the same amount of reduction in sleep, experimentally provoked during the non-migratory season (summer and winter), decreased the acquisition of components and the performance in a cognitive task (Gobes et al., 2010).

These set of studies suggest that sleep and neurogenesis are both essential to promote memory and learning, namely song learning and production. However, in birds, neurogenesis is higher when they sleep less. This negative correlation may be explained by the fact that short sleep deprivation (such as that seen in birds in the migratory season) can modulate the increase of neurogenesis, an effect verified in some experiments with rodents (Zucconi et al., 2006; Juneka et al., 2010), as will be discussed below. However, this positive effect of short sleep deprivation on neurogenesis of birds may only be seen in the migratory season due to the influence of other modulatory seasonal factors, such as sexual hormones.

Seasonal changes in hippocampal neurogenesis of rodents have been documented, however, no direct correlation with seasonal sleep patterns was established. Galea and McEwen (1999) observed higher rates of cell proliferation in adult female wild meadow voles (long-day breeders) captured during non-breeding season. However, the authors attributed these seasonal fluctuation to hormone levels (since high levels of corticosterone and estradiol are negatively related with cell proliferation) and they related these evidences with changes in spatial learning. Ormerod and Galea (2003) acclimated laboratory-reared adult male meadow voles to short- or long-photoperiods, to induce non-breeding or breeding season, respectively. They found a similar density of BrdU-labeled cells in both reproductively active and inactive males. However, 5 weeks after, the density of labeled cells was elevated only in the reproductively active males, suggesting that reproductive status regulates the survival of new cells but not cell proliferation (Ormerod and Galea, 2003).

Sakamotoa et al. (2011) hypothesized that continuous adult neurogenesis is necessary to maintain and optimize innate olfactory responses, which is essential for social communication and predator avoidance (Walton et al., 2012), cues that vary seasonally (Heth et al., 1996). Walton et al. (2012) demonstrated an increased neurogenesis in the olfactory bulb of mice exposed to short-photoperiods for 10–15 weeks, comparatively to animals exposed to long-photoperiods, which they associated to changes in olfactory behavior.

Lavenex et al. (2000) examined adult neurogenesis in the dentate gyrus of wild eastern gray squirrels at three time points: in October, at the peak of the caching season; in January, when caching is complete but squirrels are still dependent on spatial memory to locate their caches; and in June, when squirrels are not actively engaged in further caching activity. Controversially, they demonstrated that cell proliferation and total neuron number are stable throughout the year, suggesting that other factors may modulate neurogenesis in this specie.

Structural and functional seasonal plasticity are essential properties of nervous system in several species inhabiting seasonal environments (Tramontin and Brenowitz, 2000). Despite the controversial results, neurogenesis appears to be seasonally influenced in some species, and this evidence may provide clues to the role of new generated neurons. To our knowledge, there is no evidence reporting a strict correlation between hibernation, sleep and neurogenesis, since seasonal variations in neurogenesis associated with seasonal variations in sleep have not been directly addressed. This association can be established in birds in an indirect way, due to their clear sleep and neurogenesis patterns during migratory and non-migratory seasons but, in rodents, there is no consensus. Seasonal neurogenesis patterns of rodents appear to be influenced by other factors, such as an enhanced in circulating sex steroids during the breeding season or enriched environments.

## The Role of Daily Rhythms on Adult Neurogenesis

### Current Findings in Daily Changes of Neurogenesis

Sleep is a circadian rhythm (Easton et al., 2004). If sleep plays a direct role in neurogenesis (Meerlo et al., 2009), it is conceivable to expect a rhythm in cell proliferation parallel to sleep–wake cycle.

There is substantial literature on the influence of daily rhythms in cell proliferation. In two studies, for instance, using male mice, it was observed that the number of proliferating cells in the SGZ is independent of the period of day. In the first study, BrdU was administered at six equally spaced periods, during 24 h of light-dark cycle. The number of BrdU-labeled cells, analyzed 2 h after the BrdU injections, showed no significant changes throughout light-dark cycle (Kochman et al., 2006). A second study, with the same procedure but using Ki-67 as a marker, found the same evidences (van der Borght et al., 2006). In an additional experiment of this study, the authors evaluated the effects of physical activity in the number of proliferating cells. They stimulated a group of mice with a running wheel for 9 days, while a control group were placed in a standard cage. After the experimental procedure, the animals were euthanized, before or after their active period (the night of circadian cycle). Cell proliferation was significantly stimulated by physical exercise, with a strongest effect after their active phase. This effect was not observed during their resting period. Collectively, the results of these two experiments suggest that hippocampal cell proliferation is coordinated with the behavioral activity but not with the circadian rhythm (van der Borght et al., 2006).

In order to analyze the association between circadian phase and exercise in neurogenesis, Holmes et al. (2004) allowed mice free access to a running wheel for 1, 2, or 3 h per day, in three different time points: middle of the light phase; beginning and middle of the dark phase. Running activity significantly promoted proliferation, survival, and newborn neurons in the mice with access to the running wheel for 3 h during the middle of the dark phase. This evidence proposes a modulation of both the circadian phase and the amount of exercise in the influence of physical activity on neurogenesis.

In a study with adult rats (Guzman-Marin et al., 2007b), two groups of animals were BrdU-injected four times: 2, 9, 14, or 21 h after de beginning of the light phase. Animals of the experimental group lived in a complex environment, while animals of the control group were placed in standard cages. Brains samples were collected 2 h post-injections. In both groups, regardless of their daily activity (regulated by the complexity of the environment of the cages), the animals injected 9 h after the beginning of the light phase showed a significant increase of BrdU-positive cells in the SGZ. These results evidenced a daily rhythm of cell proliferation, with a proliferation peak at the end of the light period, suggesting that cell proliferation may be enhanced by sleep and other variables associated with the light phase.

Tamai et al. (2008) explored daily variations in the division of neural progenitors in the SGZ and SVZ of adult mice. They found a clear day/night variation in M-phase cells, with a significant increase during the night. This increase was correlated with an increase of newborn neurons in the SGZ during the same period. However, they found no variation in the number of S-phase progenitors across the day–night cycle. In the SVZ, no variation in the number of M-phase cells was found throughout the day–night cycle, suggesting that the influence of daily rhythms on cell proliferation may have a regional specificity (Tamai et al., 2008).

### The Controversial Data on Dentate Gyrus Neurogenesis Changes Along the Day

Most experiments with rodents reject the hypothesis that cell proliferation in the SGZ has a significant daily rhythm. Studies with mice show that proliferating cell rate more dependent on daily exercise than on circadian rhythms (Holmes et al., 2004; Kochman et al., 2006; van der Borght et al., 2006). One study with rats points to a daily rhythm in cell proliferation, with a peak in the end of their sleep period, regardless of daily activity (Guzman-Marin et al., 2007b). It is unclear, from these set of results, whether this peak is dependent on sleep, circadian rhythms or daily exercise. Differences in genetic background between rats and mice may contribute to these controversial results.

Moreover, although the SGZ is the most significant region of cell proliferation, a small amount of proliferating cells was found in the hilus (Meerlo et al., 2009). Studies with mice, that found no daily rhythms in cell proliferation in the SGZ, reported higher levels of cell proliferation in the hilus during the sleep phase (Ishida et al., 2005; Kochman et al., 2006). It is thought that neurogenesis in the hilus generates more glial cells than neurons. Thus, these results suggest a circadian influence, possibly associated to sleep, in gliogenesis of mice (Meerlo et al., 2009).

### Potential Mechanisms: the Possible Influence of Hypothalamus-Pituitary-Adrenal Axis

It has been proposed that the influence of daily rhythms on cell proliferation rate is modulated by adrenal steroid levels, which are regulated by the activation of the HPA axis (Cameron and McKay, 2001).

The HPA axis is the greatest part of the neuroendocrine system and controls several body processes. Its complex set of direct influences and feedback interactions includes the paraventricular nucleus (PVN) of the hypothalamus. The PVN has neuroendocrine neurons responsible for synthesizing and releasing vasopressin and corticotropin-releasing hormone (CRH). These two hormones regulate the anterior lobe of the pituitary gland, stimulating the secretion of adrenocorticotropic hormone (ACTH). The ACTH stimulates the adrenal cortex to synthesize and release glucocorticoid hormones (mainly cortisol in humans), which act in a negative feedback cycle on the hypothalamus and pituitary gland. The CRH secretion can be influenced by stress, physical activity, illness, sleep/wake cycle and circadian rhythm (Cameron and McKay, 2001).

The activity of the HPA axis has marked circadian effects (Ishida et al., 2005). Circadian rhythms influence cortisol secretion, through the connections between the PVN and SCN. In humans, cortisol levels reach their lowest point at midnight, starting to rise ∼2–3 h after and reaching its peak around 9 am, induced by light (Buckley and Schatzberg, 2005). The increase of glucocorticoid levels may induce a decrease in the basal rate of cell proliferation during the light phase, similarly to what happens, for instance, in the presence of stressor factors. In response to stress, the HPA axis promotes the secretion of adrenal steroids (Fuchs and Gould, 2000; Duman et al., 2001), that are negatively correlated with the hippocampal neurogenesis (Cameron and McKay, 2001). Thus, if cortisol reaches its lowest level during sleep, it is expected an increase in cell proliferation at this phase.

## Sleep Deprivation Studies

### Different Levels of Sleep Deprivation

Sleep disruption induces psychological and neurobiological changes (Goel et al., 2009), and affects a range of cognitive domains such as attention and working memory (Diekelmann and Born, 2010). To determine the role of sleep deprivation on neurogenesis, several studies used experimental paradigms to interrupt sleep of rodents for different periods of time. In experiences of total or partial sleep deprivation, wakefulness is forced through a variety of methods, such as soft handling, forced locomotion in a slowly rotating wheel or by placing rats on water-covered platforms. Effects unrelated to sleep loss (as exercise and/or stress) were controlled by the inclusion of additional groups or experimental conditions (**Table 2**).

**Table 2:** Effects of sleep deprivation on hippocampal neurogenesis.

Experimental model	Effects of sleep deprivation	Limitations	Reference
Adult male *Sprague-Dawley* rats were 48 h sleep-deprived by a disk-over-water paradigm. After, one group had 8 h of recovery sleep, while the other had more 8 h of sleep deprivation. A control group was undisturbed	Dentate gyrus cell proliferation was 39% reduced in the first group and 36% reduced in the second group	Corticosterone levels were not controlled. Cell proliferation was not analyzed by hippocampal areas	Tung et al. (2005)
Adult male *Sprague-Dawley* rats were acute (24 h) or prolonged (72 h) sleep-deprived by the small-platform method. The experimental procedure was reproduced in adrenalectomized animals	Cell proliferation was significantly reduced in the SGZ of the animals prolonged sleep-deprived. This reduction persisted by 1 and 3 weeks and it was eliminated in adrenalectomized animals	Sleep stages deprived and daily exercise were not controlled. Cell proliferation was not analyzed by hippocampal areas. The results can be influenced by low levels of corticosterone	Mirescu et al. (2006)
Male C57Bl/6 mice were acute sleep-deprived (10–12 h) by the ‘gentle handling’ method	Basal rate of cell proliferation in the SGZ was not affected	Sleep stages deprived and daily exercise were not controlled	van der Borght et al. (2006)
Treadmill sleep-deprived and treadmill control group of rats were sleep-deprived for 96 h on a treadmill that moved either for 3 s on/12 s off or for 15 m on/60 m, respectively. A cage control group was undisturbed	The number of proliferating cells in the dentate gyrus was 54% reduced in the first group comparatively with the second and 68% reduced comparatively with the control group	Sleep stages deprived, corticosterone levels and daily exercise were not controlled	Guzman-Marin et al. (2005)
An intermittent treadmill deprivation system was used to sleep-deprived rats for 96 h	Proliferation of new neurons was reduced by 50% after 96 h of sleep deprivation and 3 weeks after, mature cells with neuronal phenotype was 35% reduced	Sleep stages deprived, levels of corticosterone and daily exercise were not controlled.	Guzman-Marin and McGinty (2006)
*Sprague-Dawley* rats were parcial sleep-deprived during 12 days.	Sleep fragmentation reduced proliferating cells by 32%	Levels of corticosterone and daily exercise were not controlled. Cell proliferation was not analyzed by hippocampal areas	Sportiche et al. (2010)
Rats were sleep-restricted by drums slowly rotating for 1 day or repeatedly for 20 h/day, during 8 days	Acute sleep deprivation significantly decreased hippocampal cell proliferation in the hilus. Prolonged partial sleep deprivation decreased cell proliferation in the hilus and SGZ	Sleep stages deprived, levels of corticosterone and daily exercise were not controlled. The results can be influenced by chronic forced activity	Roman et al. (2005)
Adolescent male rats were: chronic partial sleep-deprived by slowly rotating drums; forced to walk by rotating drums at double speed; and undisturbed. Anxiety, anhedonia and HPA axis activity was assessed.	Hippocampal volume was significantly reduced in the first group but this did not significantly alter survival of newborn cells	Sleep stages deprived was not controlled. Cell proliferation was not analyzed by hippocampal areas	Novati et al. (2011)
Rats were REMS-suppressed for 4 days by a treadmill. Control animals received the same stimulus randomly during sleep stages	The proliferation of hippocampal cells was 63% reduced by REMS loss	REMS deprivation can change NREMS or waking behavior that may be essential for cell proliferation	Guzman-Marin et al. (2008)
Intact and adrenalectomized male rats were REMS-deprived for 96 h, using multiple and single-platform methods	Cell proliferation was 50% reduced in intact and adrenalectomized that received corticosterone replacement via subcutaneous minipumps	REMS deprivation can change NREMS or waking behavior that may be essential for cell proliferation. Low levels of corticosterone can promote cell proliferation	Mueller et al. (2008)
Male *Sprague-Dawley* adrenalectomized rats were REMS-deprivated by the plataform-over-water method for 4 days or exposed to constant bright light for the same time	Only REMS deprivation suppressed cell proliferation, by 50%.	REMS deprivation can change NREMS or waking behavior that may be essential for cell proliferation. Low levels of corticosterone can promote cell proliferation	Mueller et al. (2011)
Rats were sleep-deprived for 12 h, by gentle handling	Cell proliferation and total number of surviving cells increased in the SGZ after sleep deprivation, as well as 15 and 30 days after	Levels of corticosterone, sleep stages and daily exercise were not controlled. Cell proliferation was not analyzed by hippocampal areas	Zucconi et al. (2006)
Rats were sleep-deprived for 6, 12, 24, 36, or 48 h, by slowly rotating wheels	Proliferating cells in the SGZ increased significantly after 12 h of sleep deprivation but tended to decrease after 48 h of sleep deprivation.	Sleep stages and daily exercise were not controlled	Juneka et al. (2010)

### Proliferation and Survival of New Neurons in Prolonged Sleep Deprivation

Sleep has been hypothesized as a facilitator of adult hippocampal neurogenesis, since prolonged sleep deprivation of adult rodents has a negative impact on cell proliferation, survival, maturation and differentiation of new neurons in the SGZ (Mirescu et al., 2006). However, short sleep deprivation (lesser than 24 h) seems to not affect neurogenesis (Roman et al., 2005; Mirescu et al., 2006; Guzman-Marin et al., 2007a).

The effects of prolonged sleep deprivation on cell proliferation rate vary between studies and can be persistent. Tung et al. (2005) sleep-deprived two groups of adult male *Sprague-Dawley* rats for 48 h, using a disk-over-water paradigm. After, one group was allowed to sleep for 8 h, while the other group had an additional sleep deprivation period for 8 h. A control group was not sleep-deprived. After, animals were BrdU-injected and brain samples were collected 2 h later. The dentate gyrus of rats sleep-deprived for 56 h showed a reduction on cell proliferation of 36%, comparatively to animals of the control group. A similar reduction (of 39%) was observed in rats allowed to a sleep recovery for 8 h, demonstrating that the suppressive effects of prolonged sleep deprivation on cell proliferation are maintained after a sleep recovery for 8 h.

Mirescu et al. (2006) found similar evidences with adult male *Sprague-Dawley* rats. They examined the effects of acute (24 h) and prolonged (72 h) sleep deprivation on cell proliferation on the granule cell layer, marking proliferating cells with BrdU. Two hours after the injections, the number of BrdU-labeled cells of animals acute sleep-deprived did not differ from undisturbed animals. However, BrdU-labeled cells were significantly reduced in animals submitted to a prolonged sleep deprivation. This reduction remained 1 week after the injections as well as 3 weeks after, when BrdU-labeled cells should express mature neuronal nuclei.

Mice with 6–8 weeks old were sleep-deprived for 10–12 h, starting at the beginning of their sleep period. Mice of the control group were undisturbed. After sleep deprivation, corticosterone levels in plasma were similar to control mice, suggesting that the animals of the experimental group were not stressed. Mice were BrdU-injected and, 2 h later, they were euthanized. As result, the number of BrdU-labeled cells was no altered, confirming the hypothesis that the basal rate of cell proliferation in the SGZ is not affected by short sleep restriction (van der Borght et al., 2006).

In an experiment by Guzmán-Marín et al. (2003), male rats were divided into three groups: the treadmill sleep-deprived group, which was kept awake for 96 h on a moving treadmill for 3 s on/12 s off; the treadmill control group, which was kept awake for 96 h on a moving treadmill for 15 min on/60 min off; and the control group, which was undisturbed. In the first two groups, the treadmill moved the same amount of time, but it allowed sustained periods of rest to the second group. BrdU was injected 48 h after the end of the experimental procedure. As result, the number of BrdU-labeled cells in the dentate gyrus was 54% reduced in the first group, comparatively to the second group, and 68% reduced comparatively to the control group. Later, Guzman-Marin and McGinty (2006) used an intermittent treadmill deprivation system to sleep-deprived rats for 96 h. BrdU and neuron-specific nuclear antigen (NeuN) were used as markers to quantify proliferative and neurogenic processes, respectively. Cell proliferation was 50% reduced after 96 h of sleep deprivation. Three weeks after, cells with a mature neuronal phenotype were 35% reduced.

The suppression of neurogenesis by sustained sleep fragmentation has been proposed to alter cognitive functions supported by the hippocampus (Sportiche et al., 2010). These effects were furthermore predicted to persist for the same time window of the functional maturation of new neurons. To test this hypothesis, male *Sprague-Dawley* rats were sleep-fragmented for 12 days by a treadmill and compared with sleep-fragmented control group. A treadmill control group was used to evaluate whether the physical activity, alone, would affect neurogenesis. Animals of these group were placed in an identical treadmill for 12 days, which was activated during equal periods of time but only in the waking phase. A cage control group was undisturbed. Animals were injected with BrdU on day 4 and 5 after the beginning of the experimental procedure, and returned to their cages for 14 days. Thereafter, to assess cognitive performance, animals were stimulated for 5 days with a Barnes maze with a constante escape tunnel position and 2 days with a rotated escape tunnel. Then, animals were sacrificed and their samples of dentate gyrus were collected and immunolabeled for BrdU and NeuN. Sleep fragmentation reduced BrdU-labeled cells by 32% in the sleep fragmentation group, comparatively to the sleep fragmentation and the treadmill control groups. In the cognitive assessment, animals of the sleep fragmentation group demonstrated a gradual reduced in escape time, but slower than animals of the other groups. Their most used search strategy was non-spatial and random, which persisted 2 weeks after the end of the experimental procedure (Sportiche et al., 2010).

In humans, Neylan et al. (2010) determined whether insomnia severity was associated with a decrease in the volume of the hippocampal dentate gyrus. With imaging techniques, they compared the volume of hippocampal subfields of veteran age-matched men positive and negative for posttraumatic stress disorder diagnosis. Quality of sleep was assessed by the Insomnia Severity Index. As a result, higher scores on this test, indicative of worse insomnia, were correlated with reduced dentate subfields. These findings revealed an association between chronic sleep disruption and diminished volume of hippocampal subfields, suggesting a decreased neurogenesis and dendritic branching.

The effects of sleep deprivation on neurogenesis of the dentate gyrus are contradictory, including results that report a lack of specificity in antineurogenic effects of sleep loss.

Roman et al. (2005) observed the effects of sleep deprivation in different stages of hippocampal neurogenesis of rats. They sleep-deprived animals for 1 day or for 20 h per day, during 8 days, using slowly rotating drums. Addictionaly to a cage control group, a fourth group was composed, in which rats were forced to walk at double speed for half of the time, walking the same distance but being allowed to sleep. As a result, rats sleep-deprived for 1 day demonstrated a decreased cell proliferation in the hilus of the dentate gyrus. Repeated partial sleep restrition decreased cell proliferation both in the hilus and SGZ. However, a similar result was found in the fourth group, which might mean that this decrease was not a specific effect of sleep loss. In order to examine neuronal survival and differentiation, animals were injected with BrdU, NeuN and GFAP, a glial cell marker. Sleep restriction did not significantly affect the number of surviving BrdU-positive cells, as well as differentiated NeuN-positive and GFAP-positive cells. In conclusion, both prolonged or repeated partial sleep deprivation have negative effects on cell proliferation, mainly in the hilus, suggesting that sleep deprivarion may mostly decrease hippocampal gliogenesis. However, sleep deprivation did not appear to significantly affect the survival and differentiation of newborn cells.

Novati et al. (2011) divided adolescent male rats (30–61 postnatal days) in three groups: the chronic sleep restriction group, the forced activity control group, and the undisturbed control group. In the first group, rats were stimulated by a slowly rotating drum for 20 h and were allowed to sleep for 4 h per day, at the onset of their rest period. Animals of the second group were placed in a similar drum, which rotate at double of the speed for half of the time during the last 10 h of the dark phase, being allowed to sleep normally. After 1 and 4 weeks of sleep restriction, anxiety was assessed by an open field and elevated plus maze test, anhedonia was assessed by saccharin preference and HPA axis activity was evaluated. By the end of the experimental procedure, the hippocampal volume and neurogenesis were measured. Although the lack of behavioral changes, the hippocampal volume of the animals chronic sleep-deprived was significantly reduced 1 week after the experience. However, 4 weeks after the experience, this volume reduction was not reflected in changes in the survival of newborn cells, marked with BrdU, or in changes in the number of new neurons, marked with DCX. These results are not explained by high levels of corticosteroids, since the plasma levels of ACTH and corticosterone in the chronic sleep restriction group were low, and similar to other animals. Thus, these results indicate that insufficient sleep may cause reductions of hippocampal volume, however, independently of the HPA activity or neurogenesis.

### REM Sleep and NREM Sleep Deprivation

There is evidence that a reduction in cell proliferation is associated with REMS restriction, while a decrease in the differentiation of adult neurons may be associated with restrictions in both NREMS and REMS (Meerlo et al., 2009). In most studies with rodents, sleep deprivation affects indistinguishably NREMS and REMS, and only few methods cause a specific reduction in REMS (Mueller et al., 2008).

One study selectively suppressed REMS on male *Sprague–Dawley* rats for 4 days, waking animals with a treadmill only when they entered in this stage (Guzman-Marin et al., 2008). Animals of a second group received the same stimulus, randomly during REMS or NREMS. NREMS and slow wave sleep, the third stage of NREMS, did not differ significantly between these two groups. Animals of a cage control groups were maintained undisturbed. The hippocampal proliferating cells were 63% reduced in animals REMS-deprived, comparatively to animals of the second group and 82% reduced, comparatively to cage controls. However, cell proliferation was also 51% reduced in the second, group comparatively to controls.

In a similar experience, Mueller et al. (2008) REMS-deprived intact and adrenalectomized male rats for 96 h, using multiple and single-platform methods. In intact rats, proliferating cells of the dentate gyrus, marked with BrdU and Ki67, were 50% reduced, comparatively to undisturbed controls. This effect was observed in adrenalectomized rats that received continuous low-dose of corticosterone replacement via subcutaneous minipumps, but supressed in animals that received this replacement via drink water. In this group, corticosterone intake was 60% reduced, suggesting that low doses of corticosterone may upregulate cell proliferation, inhibiting the effects of sleep loss. Electroencephalogram records show that, with REMS deprivation, REMS was reduced by 95%, while NREMS was 40% reduced and slow wave sleep was reduced by 45%. This suggest that, although REMS deprivation methodology was efficient, it also partially suppressed NREMS and slow wave sleep. Later, Mueller et al. (2011) REMS-deprived adrenalectomized male *Sprague-Dawley* rats for 4 days. Other group of animals was stimulated with a constant bright light for the same time, attenuating or eliminating their daily rhythms but not affecting daily periods of REMS. Proliferating cells of the dentate gyrus, marked with BrdU, were 50% reduced by the REMS deprivation, and not affected by the constant bright light stimulation. NREMS and slow wave sleep did not differ significantly between groups. These results suggest an antineurogenic effect of REMS deprivation, and suport the hypothesis that hippocampal cell proliferation and survival may be independent of circadian rhythms (Mueller et al., 2011).

This set of results suggests that the decrease in hippocampal cell proliferation may be associated with a decrease in REMS, but does not exclude the role of NREMS and slow wave sleep. The experiment of Mueller et al. (2008) raises the the hypothesis that the disruption of NREMS and slow wave sleep or the disruption of interactions between REMS and NREMS may modulate the suppression of cell proliferation. On the other hand, the disruption of REMS may change NREMS and waking behavior, which may be essential for a normal cell proliferation. Thus, the role of NREMS on neurogenesis cannot be determined without selective deprive NREMS or slow wave sleep, which has methodological limitations due to the difficulties to sustain normal levels of REMS after NREMS deprivation.

### The Controversy: Acute Sleep Deprivation Enhance Neurogenesis

Short sleep deprivation has little negative effects on basal rates of cell proliferation and survival. Zucconi et al. (2006) sleep-deprived adult rats for 12 h, during their period of rest. Rats were BrdU-injected 4 h before and 2 h after sleep deprivation, and sacrificed at that moment, 15 or 30 days later. The results showed that 12 h of sleep deprivation significantly increased cell proliferation and survival in the dentate gyrus immediately after sleep deprivation, as well as 15 and 30 days later, comparatively with non-sleep-deprived animals. No changes were found in the SVZ, indicating that short sleep deprivation may be selectively related to hippocampal neurogenic signals.

Juneka et al. (2010) observed the effects of different periods of sleep deprivation on cell proliferation in the SGZ of adult rats. Animals were sleep-deprived for 6, 12, 24, 36, or 48 h and BrdU was administered 2 h before the end of sleep deprivation. The number of BrdU-labeled cells increased significantly after 12 h of sleep deprivation, and decreased after 48 h of sleep deprivation, comparatively to non-sleep-deprived controls. Proliferating cells were marked with Ki-67 or with proliferating cell nuclear antigen (PCNA). Sleep deprivation for 12 h did not alter immunolabeling for Ki-67, as well as PCNA and corticosterone levels. Immunoreactivity for Ki-67 and PCNA can mark cells in all phases of the cell cycle of the hippocampus of rats (∼25 h), while BrdU only labels cells in S-phase (∼9.5 h). These contradictory results indicate that 12 h of sleep deprivation might have affected the dynamics of the cell cycle. To test this hypothesis, rats of a different group were BrdU-injected 10 h before the end of 12 h of sleep deprivation. The results point to an acceleration of cell division of hippocampal progenitors, suggesting that short sleep deprivation increase the production of hippocampal progenitor cells by temporarily accelerating the cell cycle.

## Potential Mechanism

The underlying mechanisms of the negative effects of sleep deprivation on different stages of adult neurogenesis are unknown. It has been hypothesized that these effects can be indirectly mediated by stress and their hormones, particularly by glucocorticoids. For instance, sleep deprivation of rats, with the small-platform method, significantly increased their leves of corticosterone and significantly decreased their cell proliferation. This experiment was replicated in adrenalectomized mice, which produce low levels of corticosterone, and the decrease of cell proliferation in the dentate gyrus was completely eliminated (Mirescu et al., 2006).

These results are controversial and contrast with recent studies described above, in which antineurogenic effects of sleep deprivation on the hippocampus were maintained with low levels of corticosterone (van der Borght et al., 2006; Guzman-Marin et al., 2008; Mueller et al., 2008; Novati et al., 2011). Furthermore, while high levels of corticosterone suppress cell proliferation, low levels of corticosterone promote cell proliferation (Cameron and Gould, 1994).

Adult neurogenesis is regulated by several molecular factors, including trophic factors, cytokines, hormones and neurotransmitters (Cameron and McKay, 2001; Kempermann, 2002). Many of these factors are affected by sleep deprivation and this may provide a link between insufficient sleep and reduced hippocampal neurogenesis. For instance, serotonin stimulate hippocampal neurogenesis due to the serotonin-1A receptor action (Radley and Jacobs, 2002; Banasr et al., 2004). Serotonergic activity is relatively low during sleep, which may not explain the suppressive effect of sleep deprivation in neurogenesis. However, this lower serotonergic activity during sleep may be necessary for the normal serotoninergic activity during wakefulness and, consequently, it may be important for a waking experience effects in neurogenesis. In rats, chronic sleep deprivation cause a reduction in the sensitivity of the serotonin-1A receptor system (Novati et al., 2008), which is not evident in short sleep deprivation. Similarly, several evidences suggest that short sleep deprivation does not appear to affect cell proliferation, while chronic sleep deprivation decrease cell proliferation in the hippocampus.

Insulin-like growth factor (IGF)-1 is one of several growth factors known as neurogenesis promoters (Trejo et al., 2001). Prolonged sleep deprivation in rats showed lower IGF-1 binding (Everson and Crowley, 2004). The Brain-derived neurotrophic factor (BDNF) also facilitates hippocampal neurogenesis (Scharfman et al., 2005; Guzman-Marin et al., 2006). The hippocampal expression of BDNF was decreased after 8 and 48 h of sleep deprivation (Guzman-Marin et al., 2006). This decrease is associated with REMS suppression, what may be an important evidence, given the association between the suppression of hippocampal cell proliferation and REMS loss (Guzman-Marin et al., 2008).

Growth hormone (GH) could also be related with neurogenesis regulation. A recent study showed that GH administration strongly promotes adult cell proliferation in the dentate gyrus of rats, and protects the hippocampal neuronal precursors of the negative effects of chronic sleep deprivation (Irwin et al., 2006; García-García et al., 2011). The protective role of GH could have clinical relevance, since GH replacement therapy appears to improve both mood and sleep quality (Mahajan et al., 2004; Haack et al., 2007), effects that might be related to the neurogenesis in the hippocampus.

A decrease of cell proliferation after sleep deprivation can be associated to enhanced levels of pro-inflammatory cytokines, interleukin (IL)-6 and tumor necrosis factor (TNF-a). There are evidences that both IL-6 and TNF-a are increased after chronic sleep deprivation (Irwin et al., 2006; Haack et al., 2007), and IL-6 plasma levels are enhanced in patients with insomnia (Burgos et al., 2006). *In vitro,* exposure to IL-6 and TNF-a diminishes cell proliferation and, *in vivo*, they can modulate the damaging effects of neuroinflammation in hippocampal neurogenesis (Monje et al., 2003).

**FIGURE 1:**
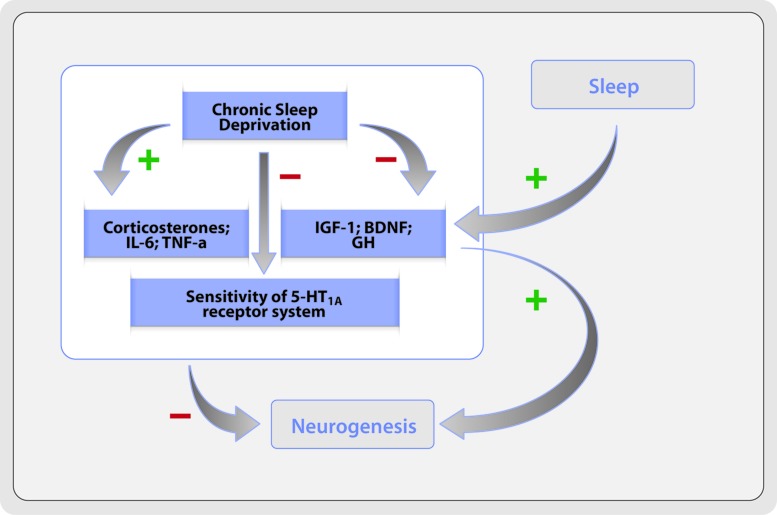
**Potential mechanisms of anti-neurogenic effects of chronic sleep deprivation**.

The mechanism by which prolonged sleep deprivation affects adult neurogenesis may imply a complex group of interacting factors (**Figure 1**), which may, in fact, affect selectively different stages of neurogenesis process (Meerlo et al., 2009).

## Can Sleep Deprivation Affect Neurogenesis in SVZ, or Other Adult Neurogenic Areas?

Most studies on the association between adult neurogenesis and sleep were focused on the dentate gyrus, excluding the SVZ. This can be explain by a greater interest in the hippocampus than in the olfactory bulb, the area to where migrate the majority of newborn cells generated in the SVZ (Meerlo et al., 2009).

Mirescu et al. (2006) studied the effects of 3 days of sleep deprivation in the SVZ and found no changes in cell proliferation, while it was significantly decreased in the SGZ, suggesting that effects of sleep deprivation may be regionally selective (Mirescu et al., 2006). Even within the dentate gyrus, some regional variations may be found. The effects of sleep deprivation appear stronger in ventral than dorsal region of the dentate gyrus (Tung et al., 2005). It may be important to clarify these findings since different regions of the hippocampus have differente functions. Selective lesions in rodents revealed that the dorsal hippocampus seems to be responsible for certain forms of learning and memory, namely spatial learning, while the ventral hippocampus seems to be associated to regulation of emotional behavior (Moser and Moser, 1998).

## Other Interacting Aspects of Sleep and Neurogenesis

Several studies have focused on the possible role of new neurons in the hippocampal functions, since hippocampus is the major component of cognitive-limbic system and important to cognition processes, like learning and memory (Moser and Moser, 1998). Additionally, the hippocampus establishes reciprocal connections with several brain areas, such as the amygdala and prefrontal cortex, which regulate the emotionality. It has been proposed that antineurogenic effects of sleep deprivation act as a mediating factor on cognitive and mood deficits (Jacobs et al., 2000; Sportiche et al., 2010).

### Learning and New Memory Formation

Hippocampus-dependent learning and memory are associated with an increase of hippocampal neurogenesis (Dobrossy et al., 2003), while learning deficits are associated with a decrease (Leuner et al., 2006). In addition, several conditions that decrease cell proliferation on the dentate gyrus (such as acute and cronic stress, increased levels of circulating corticosteroids, aging and opiates) also damage the hippocampus-dependent learning. Moreover, conditions that enhance hippocampal cell proliferation (such as increased environmental complexity, physical exercise, and estrogen levels) are associated with an increase in learning and new memories formation (Gross, 2000). Finally, experimental tasks that stimulate hippocampal-dependent learning seem to contribute to a longer survival of newborn neurons in the hippocampal dentate gyrus (Gould et al., 1999).

Sleep appears to play a role in learning and new memories formation, and its deprivation disrupts these two cognitive functions. This evidence suggests that sleep may support learning and memory by promoting survival, maturation and functional integration of new hippocampal neurons, under an unexplored mechanism (Meerlo et al., 2009).

A study with rats examined if the effects of sleep deprivation on hippocampal-dependent learning was related to a decrease in the survival of new cells. Two groups of animals were stimulated in a water maze for 4 days, in a hippocampal-dependent spatial task (a submerged and invisible platform) or in a non-hippocampal-dependent spatial task (a visible platform). After each training day, on group of animals were kept awake for the first 6 h of its normal rest time. Animals were BrdU-injected 1 week before the beginning of the training. During the experience, the generated cells a week before the training should be mature and incorporated in the hippocampal network. Rats trained on hippocampal-dependent spatial task had a pronounced increase of newborn cells survival. However, this effect was suppressed in animals submitted to the same task but sleep-deprived. In the sleep-deprived group spatial learning was impaired but, surprisingly, non-spatial learning was improved. In both groups, fully rested animals applied a spatial strategy in both tasks, which interfered with the performance in the non-spatial task. In sleep-restricted group, this spatial strategy was eliminated, and animals used only non-spatial information, improving non-spatial performance. These findings suggest that sleep loss altered behavioral strategies and reversed neurogenic effects of hippocampus-dependent learning (Hairston et al., 2005)

In humans, imaging studies have confirmed the role of sleep in the hippocampal functions, particularly in learning and memory (Peigneux et al., 2004; Orban et al., 2006). In addition, the cognitive performance in patients with chronic insomnia is disturbed (Backhaus et al., 2006; Nissen et al., 2006) and imaging techniques of these patients showed a significant reduction in hippocampal volume (Riemann et al., 2007). Reduced adult neurogenesis in humans, caused by sleep disturbance, may be a limited explanation to justify this decreased volume, but may contribute to this phenomenon (Meerlo et al., 2009).

According to some models, new memories are formed due to a structural remodeling of existing synapses and due to an adaptation of the synaptic strength in the existing circuits. Sleep, after the initial learning, appears to contribute to this process through the neural repetition and reactivation of neural plasticity process (Graves et al., 2001; Stickgold, 2005; Stickgold and Walker, 2005). This synaptic plasticity dependent of sleep can involve both the existing neurons and the maturation and strengthening of synapses of new neurons.

### Mood Regulation

Recent evidences showed that hippocampal neurogenesis is reduced in animal models of depression. Furthermore, antidepressant treatment promotes neurogenesis (Zucconi et al., 2006). This raises the hypothesis that changes in the hippocampal neurogenesis may mediate emotional and cognitive deficits observed in mood disorders (Jacobs et al., 2000; Sahay and Hen, 2007).

Sleep disruption has been associated with mood regulation deficits, however, by an unknown mechanisms. In has been hypothesized that chronic sleep deprivation contributes to the etiology of depression by inhibits cell proliferation and hippocampal neurogenesis. Studies with rodents have shown that prolonged sleep deprivation progressively leads to physiological and neurobiological modifications similar to those experienced in patients with major depressive disorder (MDD; Meerlo et al., 2009).

### Pharmacology Perspective: Hypnotic Drugs and Neurogenesis

Sleep deprivation appears to suppress neurogenesis. Thus, it would be conceivable that hypnotic drugs, by improve the quality of sleep, could have a neurogenic effect. To test this hypothesis, Takase et al. (2009) administered Zolpidem (5, 10, or 20 mg/kg), a non-benzodiazepine hypnotic drug, in young and old male *Sprague-Dawley* rats (**Table 3**). Zolpidem was ingested twice daily, at the onset and at the middle of the rest phase, for 2 days (acute administration) or 21 days (chronic administration). Acute ingestion significantly suppressed cell proliferation in the aged animals. In both groups, cell proliferation supression was higher in the hilus than in the SGZ. The lower dose of Zolpidem reduced neurogenesis by 25% in the dentate gyrus of young animals and the higher dose had no significative effects. The authors argued that the chronic administration of hypnotic drugs in young animals may cause a disruption of normal sleep, which may be related to a reduction in cell survival. In aged animals, the low dose increased cell survival in the SGZ by 11% and decrease cell survival in the hilus by 6%. In aged animals, hypnotic drugs may improve the speel quality (typically disturbed at this age), causing a benefical effects on neurogenesis. However, even in aged group, chronic ingestion of Zolpidem had little or no effect on cell proliferation in both groups of rats, suggesting little benefic effects of hypnotic drugs on neurogenesis (Takase et al., 2009).

**Table 3:** Effects of hypnotic and antidepressant drugs on neurogenesis.

Drug	Effects on neurogenesis	Reference
Zolpidem (hypnotic drug) was administered in aged and young adults rats twice daily, at the onset and middle of the rest phase, for 2 days (acute study) or 21 days (chronic study)	Acute administration produced a suppression on cell proliferation in the aged (30–40%) and young adults (10–15%), larger in the hilus than in the SGZ. Chronic administration produced a small reduction of cell survival in the SGZ of young animals and a slight increase in aged animals	Takase et al. (2009)
Modafinil or caffeine (psychostimulant drugs) were administered in rats total sleep-deprived for 2 days	Prevented decline in neuronal proliferation and differentiation after sleep deprivation	Sahu et al. (2013)
Different classes of antidepressants (MAOI; SSRI; TCA) and electroconvulsive seizure were tested in adult male *Sprague-Dawley* rats	Chronic administration of electroconvulsive seizures enhance proliferating cells by 50%, while different classes of antidepressant increased proliferating cells by 20–40%	Malberg et al. (2000)
A non-antidepressant psychotropic drug (Haloperidol) was tested in adult male *Sprague-Dawley* rats	Chronic administration did not significantly alter the number of BrdU-positive cells	Malberg et al. (2000)
The number of cells in the dentate gyrus were compared between *postmortem* non-psychiatric controls, untreated MDD patients, and MDD patients treated with SSRIs or TCAs	MDD treated patients had a increase in neuronal progenitors and a larger dentate gyrus volume. Dividing cells were greater in MDD patients treated with TCAs. The increase of neuronal progenitors and dividing cells was localized on the rostral dentate gyrus.	Boldrini et al. (2009)

Psychostimulant drugs, such as Modafinil or caffeine, administered in rats sleep-deprived for 2 days, prevents the decrease of proliferating and differentiating cells, marked with BrdU and DCX, respectively (Sahu et al., 2013), opposing to the suppressive effects of the sleep deprivation in the cell proliferation and differentiation.

A major finding in the pharmacological perspective of neurogenesis was the discovery that antidepressant drugs increases hippocampal neurogenesis (**Table 3**). Thus, stimulating adult hippocampal neurogenesis may be a new drug target or mechanism of antidepressants, to reach its therapeutic effects (Takase et al., 2009).

Several studies had been demonstrating the stimulating effects of multiple classes of antidepressants on hippocampal neurogenesis in a chronic, but not acute, time course (Boldrini et al., 2009). A set of studies demonstrated that chronic antidepressant treatment can upregulate the expression of BDNF in the hippocampus (Nibuya et al., 1995), which seems to stimulate adult cell proliferation, differentiation and survival *in vitro* and *in vivo* (Takahashi et al., 1998). Addictionaly, chronic antidepressant treatment can oppose to the downregulation of the hippocampal BDNF expression caused by stress (Nibuya et al., 1995).

In a study of Malberg et al. (2000) with adult male *Sprague-Dawley* rat, it was investigated the effect of different classes of antidepressants and electroconvulsive seizure treatment on hippocampal neurogenesis, using BrdU as a marker. Chronic treatment with electroconvulsive seizures increased the BrdU-positive cells in the dentate gyrus by 50%, while chronic administration (14 or 28 days) of chemical antidepressants – a monoamine oxidase inhibitor (MAOI); a selective serotonin reuptake inhibitors (SSRIs) and a tricyclic antidepressants (TCAs) – increased the BrdU-positive cells by 20–40% (Malberg et al., 2000). Acute administration (1 or 5 days) of a SSRI did not change the number of BrdU-positive cells, suggesting that chronic, but not acute, antidepressant treatment enhance BrdU-positive cells in the hippocampus, consistently with the time window of their therapeutic action (Duman et al., 1997).

To examine the specificity of the neurogenic effects of antidepressants on neurogenesis, the influence of Haloperidol, a non-antidepressant psychotropic drug, on neurogenesis was evaluated. As a result, chronic administration of Haloperidol did not significantly alter the number of BrdU-positive cells (Malberg et al., 2000).

Boldrini et al. (2009), using post mortem tissue samples, determined the anatomical location of neural progenitor cells with Nestin-IR and proliferating cells with Ki-67 in the dentate gyrus of non-psychiatric controls, untreated MMD patients, MDD patients treated with SSRIs or TCAs in the past 3 months before death. Patients treated with SSRIs and TCA evidenced a increase in neuronal progenitor cells than untreated MDD patients and controls. Dividing cells number were higher in MDD patients treated with TCAs than in untreated MDD patients, patients treated with SSRIs, and controls. Treated patients had a larger dentate gyrus volume, comparatively to untreated MDD patients or controls and this increase of neuronal progenitors and dividing cells was localized in the rostral dentate gyrus (Boldrini et al., 2009).

The neural mechanisms underlying the therapeutic effects of antidepressants still unknown, but the upregulation of growth factors and neurogenesis may be involved. Warner-Schmidt and Duman (2007) demonstrated that VEGF is an essential mediator of the neurogenic and behavioral actions of antidepressants, by two high-affinity receptor tyrosine kinases (Flk-1 and Flt-1). Male *Sprague-Dawley* rats were and treated with electroconvulsive seizures. Animals of the control group were handled identically, but received no shock. A SSRI, a TCA, or saline was injected once or twice daily. As result, VEGF was identified as a key mediator of the actions of two classes of antidepressants and it was induced by the SSRI, the TCA and by the electroconvulsive seizure, in a time window consistent with the time window of cell proliferation and maturation and the time window of the therapeutic action of antidepressants (Warner-Schmidt and Duman, 2007).

## Future Studies

The current knowledge on adult neurogenesis comes from laboratory studies with animal models. However, adult neurogenesis has been demonstrated in humans (Eriksson et al., 1998), despite the methodological limitations of measure new generated neurons in live participants.

Recent developments of magnetic resonance imaging techniques are promising and opens the possibility to study neurogenesis in humans under several experimental conditions and to study of the association between different pathologies and neurogenesis (Shapiro et al., 2006; Manganas et al., 2007). Furthermore, imaging techniques could ultimately provide an opportunity to study the association between human sleep deprivation and neurogenesis, as well as it has been done in rodent models.

Considering all studies with animal models discussed throughout this review, there are several controversial and contradictory results. Results obtained with some species and under certain experimental conditions can not be replicated in other species, since neurogenesis and sleep regulation may be conditioned by the individual and genetic features of each specie. Thus, studies of neurogenesis with animal models need to be extended to produce more robust results on the underlying mechanisms of the modulatory effects of sleep on neurogenesis.

More studies are needed to determine the antineurogenic effects of sleep deprivation on different hippocampal areas, given the evidence of a functional differentiation of different hippocampal subfields. It is also necessary to explore the effects of sleep deprivation on neurogenesis of other brain areas, which could be useful to understanding the functional outcome of neurogenesis. The effects found in the basal rate of cell proliferation in the REMS *versus* NREM deprivation also need to be replicated to reach more robust conclusions.

The neurogenic link between sleep, learning, hippocampus-dependent memory and mood disorders need to be clarified, and the functional relevance of increased neurogenesis caused by antidepressant treatments need to be explored with clinical studies in humans. It is necessary to understand if neurogenesis is related with a behavioral and molecular response to antidepressant treatments in MDD patients. Finally, the negative hypnotic effects of neurogenesis need to be clarified and tested in humans, since they can have unknown side effects in human adult neurogenesis, with repercussions to other cognitive and emotional functions.

## Conclusion

The knowledge of neurogenesis in adult mammals has significantly progressed over the last decade (Arias-Carrión et al., 2007; Yuan and Arias-Carrion, 2011; Yuan et al., 2014). The decline of the belief that neuronal neurogenesis ends at puberty seems to be part of the recognition of the plasticity of adult brain and its structural modulation based on experience (Gross, 2000). It is now accepted that adult neurogenesis actively occurs throughout life in the SVZ and SGZ of the hippocampal dentate gyrus, appearing to be limited or absent in others areas of the CNS. However, after a pathologic stimulation, such as brain injury, neurogenesis may occur in non-neurogenic areas (Arias-Carrión et al., 2007; Yuan and Arias-Carrion, 2011).

While studies on adult human neurogenesis await the development of *in vivo* imaging techniques, data from animal models suggests that newly generated neurons in adulthood may be involved in hippocampal plasticity.

Chronic sleep disruption decreases adult neurogenesis in rodents. However, short sleep deprivation seems to enhance cell proliferation and survival. The chronic effects of sleep deprivation may impair the hippocampal integrity and may ultimately lead to cognitive dysfunction, contributing to the development of mood disorders and other pathologies. Whether adult neurogenesis plays a role in such modulation is yet to be determined. Targeting adult neurogenesis during sleep disorders might therefore to provide one interesting approach to reverse the potential disruption of chronic sleep disorders on cognitive and emotional function.

## Conflict of Interest Statement

The authors declare that the research was conducted in the absence of any commercial or financial relationships that could be construed as a potential conflict of interest.
